# Response to Comment on: “Randomized Controlled Trial of Surgical Rib Fixation to Nonoperative Management in Severe Chest Wall Injury”

**DOI:** 10.1097/AS9.0000000000000389

**Published:** 2024-02-27

**Authors:** David E. Meyer, Lillian S. Kao

**Affiliations:** * From the Department of Surgery, McGovern Medical School at UTHealth Houston, Houston, TX.

We very much appreciate Dr. Tomioka’s interest in our recently published study: “Randomized Controlled Trial of Surgical Rib Fixation Versus Nonoperative Management in Severe Chest Wall Injury.”^1^ We agree that existing protocols regarding the use of surgical stabilization of rib fractures (SSRFs) in patients without an unstable chest wall injury, especially in the absence of respiratory failure requiring mechanical ventilation, should be reevaluated. We also agree that further research will be necessary to better define the role of SSRF in chest wall injury. There is an urgent need for large, high-quality, multicenter randomized controlled trials in this field. If there is an expectation that certain high-risk subgroups might experience a differential treatment effect, future studies should be powered to detect these differences.

As Dr. Tomioka points out, there may be pathologic dissimilarities between the severe chest wall injury criteria enumerated in the trial (ie, 5 or more consecutive fractures, a radiographic flail segment, or one or more bicortically displaced fractures). Regardless, each of these criteria has been used or advocated by other studies as an indication for SSRF.^2–4^ Furthermore, multiple consecutive rib fractures are not necessarily a better target for stabilization than 1 or 2 bicortically displaced fractures. In fact, there is increasing evidence to suggest that displaced fractures present better targets for surgical intervention. This concept is demonstrated in Dehghan et al’s^5^ recent multicenter trial as well as our own study where patients with displaced fractures showed a more favorable differential treatment effect. Despite the liberal inclusion criteria, more than 90% of the patients in each group had 5 or more consecutive fractures, and two-thirds of patients had more than one severe chest wall injury criterion.

**FIGURE 1.**
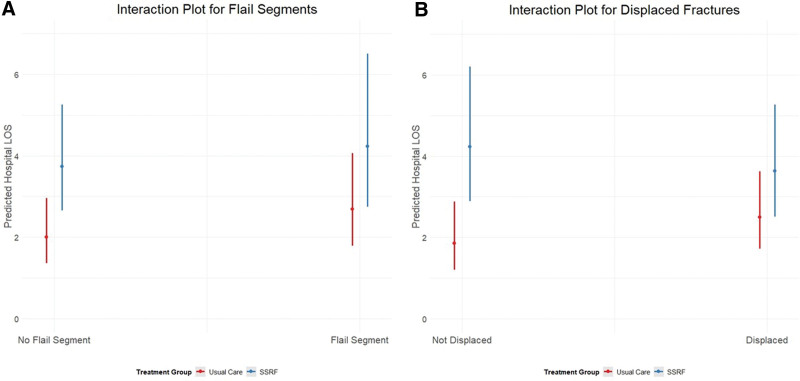
Interaction plots of SSRF in the 2 a priori subgroups: (A) patients with radiographic flail segments, and (B) patients with displaced fractures. Tests for interaction showed that SSRF patients with displaced fractures had length of stay outcomes that were more similar to (but not better than) their Usual Care counterparts. This differential treatment effect was not observed in patients with radiographic flail segments.

As suggested by Dr. Tomioka, subgroup analyses may provide insights into heterogeneity of treatment effect. As the study was powered only for the primary outcome (and underpowered at that, due to the lower-than-expected enrollment), any subgroup analysis would be hypothesis generating at best. Moreover, randomization was not stratified based on injury pattern such that subgroup differences occurring by chance could lead to incorrect conclusions. However, recognizing that flail segments and bicortically displaced fractures may respond differently to SSRF, we specified these subgroups a priori for additional analysis. As described, tests for interaction showed that SSRF patients with displaced fractures had length of stay outcomes that were more similar to (but not better than) their Usual Care counterparts (*P* = 0.142). While not included in the original manuscript, subgroup interaction plots are provided here for review (Fig. 1).

As noted by Dr. Tomioka, routine use of regional analgesia in the SSRF group at the time of surgery was avoided because it would introduce systematic bias. However, regional analgesia was offered to all patients who had severe pain that was refractory to oral therapy. As reported, 7% of Usual Care patients and 17% of SSRF patients received regional analgesia. Because the use of regional analgesia was similar between the randomized groups, any benefit would be expected to be balanced.

The authors also agree that long-term and patient-centered outcomes are important to measure in any SSRF research. This is why we were careful to include patient-centered outcomes and health status measurements up to 6 months following injury. As might be expected, the greatest differences in these outcomes were seen at 1 month after injury. By 3 months of follow-up, outcomes were similar between groups. This is not surprising, since fractured ribs might reasonably be expected to heal on their own by 8 to 12 weeks. Long-term outcomes remained similar at 6 months after injury. Though not measured, the authors do not expect that these results would diverge after 6 months. If there is interest in outcomes beyond 6 months following injury, they should be measured in future trials.

Finally, we wish to emphasize that the trial was not designed to be completely pragmatic given the lack of evidence for the intervention in the population studied. Nonetheless, the trial provides the least biased estimates of treatment effect for an increasingly used intervention, SSRF, in a broad population of patients. These estimates can be used to design multicenter, pragmatic trials that incorporate adequately powered preplanned subgroup analyses and longer-term outcomes. Thank you again to Dr. Tomioka for his insights and for being a powerful advocate for thoughtful design in future randomized trials of SSRF.
